# Identification of molecular subtypes based on inflammatory response in lower-grade glioma

**DOI:** 10.1186/s41232-022-00215-9

**Published:** 2022-10-01

**Authors:** Wanzun Lin, Jing Gao, Haojiong Zhang, Li Chen, Xianxin Qiu, Qingting Huang, Jiyi Hu, Lin Kong, Jiade J. Lu

**Affiliations:** 1 Department of Radiation Oncology, Shanghai Proton and Heavy Ion Center, Fudan University Cancer Hospital, 4365 Kangxin Rd, Shanghai, 201321 China; 2 Shanghai Key Laboratory of Radiation Oncology (20dz2261000), Shanghai, China; 3 Shanghai Engineering Research Center of Proton and Heavy Ion Radiation Therapy, Shanghai, China; 4 Department of Radiation Oncology, Shanghai Proton and Heavy Ion Center, Shanghai, 201321 China

**Keywords:** Inflammatory response, Lower-grade glioma, Molecular subtypes, Prognosis, Tumor microenvironment

## Abstract

**Background:**

Inflammatory response is an important characteristic affecting prognosis and therapeutic response in lower-grade glioma (LGG). However, the molecular subtypes based on inflammatory response are still under exploitation.

**Methods:**

The RNA sequencing, somatic mutation, and corresponding clinical data from 1205 LGG patients were obtained from the TCGA, CGGA, and Rembrandt cohorts. Consensus clustering was performed to identify molecular subtypes associated with inflammation. Prognosis, clinicopathologic features, immune cell infiltration, and somatic mutation profile were compared among these inflammation-associated subtypes.

**Results:**

Our results demonstrate that LGG could be categorized into inflammation-, low, -mid, and -high subtypes with distinct clinicopathologic features, prognostic and tumor microenvironment. We established that this categorization was reproducible, as well as predictable. In general, inflammation-high subtype presents a dismal prognosis with the immunosuppressive microenvironment and high frequency of oncogene mutation. Inversely, inflammation-low subtype was associated with the most favorable clinical outcomes with the immunoreactive microenvironment among three subtypes. Moreover, we develop and validate an inflammation-related prognostic model, which shows strong power for prognosis assessment.

**Conclusion:**

In conclusion, we established a novel glioma classification based on the inflammation subtype. This classification had significant outcomes for estimating the prognosis, as well as the tumor microenvironment.

**Supplementary Information:**

The online version contains supplementary material available at 10.1186/s41232-022-00215-9.

## Introduction

Inflammation is an ancient evolved biological process that combines the activation, recruitment, and activity of innate and adaptive immune cells [1, 2]. The precise role of inflammation in the occurrence, progression, and therapy of cancer has gained much research interest. It has been widely established that inflammation may perform a substantial function in carcinogenesis at all stages [3]. Acute inflammation increases cancer cell death by activating an antitumor immune response, but persistent inflammation induced by treatment enhances resistance against treatment and the progression of cancer [4, 5]. In addition, inflammation is associated with the clinical outcome, especially with immunotherapy, an auspicious therapeutic strategy for cancer treatment [6].

Glioma has been identified as the most prevalent primary malignancy located in the central nervous system, whose features include unfavorable proliferation as well as invasion of tumors [7]. The tumor microenvironments (TME) of grades II and III gliomas vary significantly from each other, despite the fact that these tumors are typically considered as diffuse lower-grade gliomas (LGGs) in general [8]. The neuroinflammation-enriched tumor microenvironment is considered as one of the important defining features of high-grade glioma and is identified as a significant factor contributing to the complexity and lethality [9]. The function of inflammatory mediators is critical in the establishment of an immunosuppressed microenvironment, resulting in the increased proliferation, invasion, and preservation of high-grade glioma cells’ stemness [10].

Multiple molecular subtypes of glioma have been identified, with the most notable being IDH mutations and the 1p/19q deletion [11]. We hypothesized that molecular subtypes classified by inflammatory response may also produce distinct clinicopathologic features, prognostic and tumor microenvironment. This study aimed to (i) identify molecular subtypes based on inflammatory response in LGG; (ii) evaluate the prognostic value, antitumor immunity, and tumor microenvironment associated with these subtypes; and (iii) construct and validate the inflammation-related prognostic model.

## Materials and methods

### Datasets

A sum of 509 LGG patients was included in the study, and their RNA sequencing, somatic mutation, and matching clinical information were acquired from the TCGA database (https://portal.gdc.cancer.gov/). To serve as a validation set, comparable data were obtained for the 121 LGG patients in the Rembrandt cohort (https://www.ncbi.nlm.nih.gov/geo/query/acc.cgi?acc=GSE108474) and the 575 LGG patients in the CGGA cohort (http://www.cgga.org.cn/) [12–14]. The clinical information of LGG patients was provided in the Additional file 1.

### Integration of protein-protein interaction (PPI) network

A network for protein-protein interactions was constructed utilizing the STRING database. Cytoscape (https://cytoscape.org/) is a free and open-source software platform that is extensively used to visualize sophisticated networks and merge them with any type of attribute data. A network for PPI was constructed, and the interaction connections of important genes in inflammation-linked genes were examined utilizing Cytoscape.

### Consensus clustering

Consensus clustering was performed to identify molecular subtypes associated with inflammation via the “ConcensusClusterPlus” package in R software. Subsequently, the optimum cluster numbers between *k* = 2 and 10 were identified, after which the procedure was repeated 1000 times to ensure that the findings were robust and reproducible. A cluster map was created using the pheatmap function in the R software.

### Principal component analysis

In order to examine the transcriptional patterns of the various inflammatory subtypes, principal component analysis (PCA) was used. It was necessary to import the gene names together with the matching sample data and level of expression. Subsequently, the analysis was carried out by the “limm” package utilizing the princomp function, and the findings were presented with the aid of “ggplot2” package in the R software.

### Single-sample gene-set enrichment analysis (ssGSEA)

The ssGSEA analysis was used to quantify the inflammatory response score of each LGG sample and was completed using the “GSVA” and “GSEABase” packages in R. Gene signature for the inflammatory response was obtained from gene-set enrichment analysis (HALLMARK_INFLAMMATORY_RESPONSE), and the gene list is provided in Additional file 2.

### Calculation of the immune cell type fractions

CIBERSORT was used to measure the 22 different types of immune cells infiltration in each LGG sample. In the CIBERSORT platform (https://cibersort.stanford.edu/), a leukocyte gene matrix containing 547 genes was employed to distinguish 22 immune cells, which included eosinophils, memory B cells, neutrophils, T cells CD4 naïve, activated mast cells, activated CD4 memory T cells, resting dendritic cells, T cells regulatory (Tregs), macrophages: M0, M1, and M2, monocytes, NK cells activated, T cells gamma delta, T cells follicular helper, NK cells resting, resting CD4 memory T cells, activated dendritic cells, T cells CD8, resting mast cells, naive B cells, neutrophils, and plasma cells [15].

### Somatic mutation analysis

TCGA GDC Data Portal was used to obtain “maf”-formatted somatic mutation data for each LGG sample (VarScan2 Variant Aggregation and Masking; https://portal.gdc.cancer.gov). Subsequently, the “Maftools” function in R software was used to create “waterfall” charts, which helped to visualize and summarize the altered genes of the three LGG subtypes and abnormal signaling pathways.

### Creation of the inflammation prognostic signature

A LASSO cox regression analysis was used to generate the particular coefficient factors for each correlation among the inflammatory-related genes that were found to have significance during the univariable Cox regression analysis. LASSO is a regression analysis method that performs both variable selection and regularization to improve predictive accuracy and the interpretability of the resulting statistical model. Hence, LASSO cox regression is an excellent option for the development of prognostic models on the basis of gene expression profiles.

Survminer and survival packages for R were used to conduct Kaplan-Meier analysis on the survival data for the high- and low-risk cohorts, and the results were compared.

### The single-cell RNA sequence (scRNA-seq) analysis

In order to examine scRNA-seq data collected from the GSE70630, the tumor immune single-cell hub (TISCH) was employed [16]. TISCH is a single-cell RNA-seq data source that puts an emphasis on th TME and provides specific annotation of cell types at the single-cell level, allowing for TME investigation across diverse malignancies [17].

### Statistical analysis

The survival and survminer modules in R were utilized to perform Kaplan-Meier analysis on patients’ overall survival (OS) and provide a comparison between various groups. The Kruskal-Wallis test or Wilcoxon signed-rank test was used to determine if there were any differences between the subtypes. To explore relevant predictive markers, we used the univariate Cox analysis. With the assistance of the survivalROC R package, an analysis of the ROC curve was carried out to determine the accuracy of the risk model in anticipating patients’ OS. The R software (version: 4.1.0) was used for all of the statistical analyses.

## Results

### Consensus clustering identified three inflammation-based subtypes

**Fig. 1:**
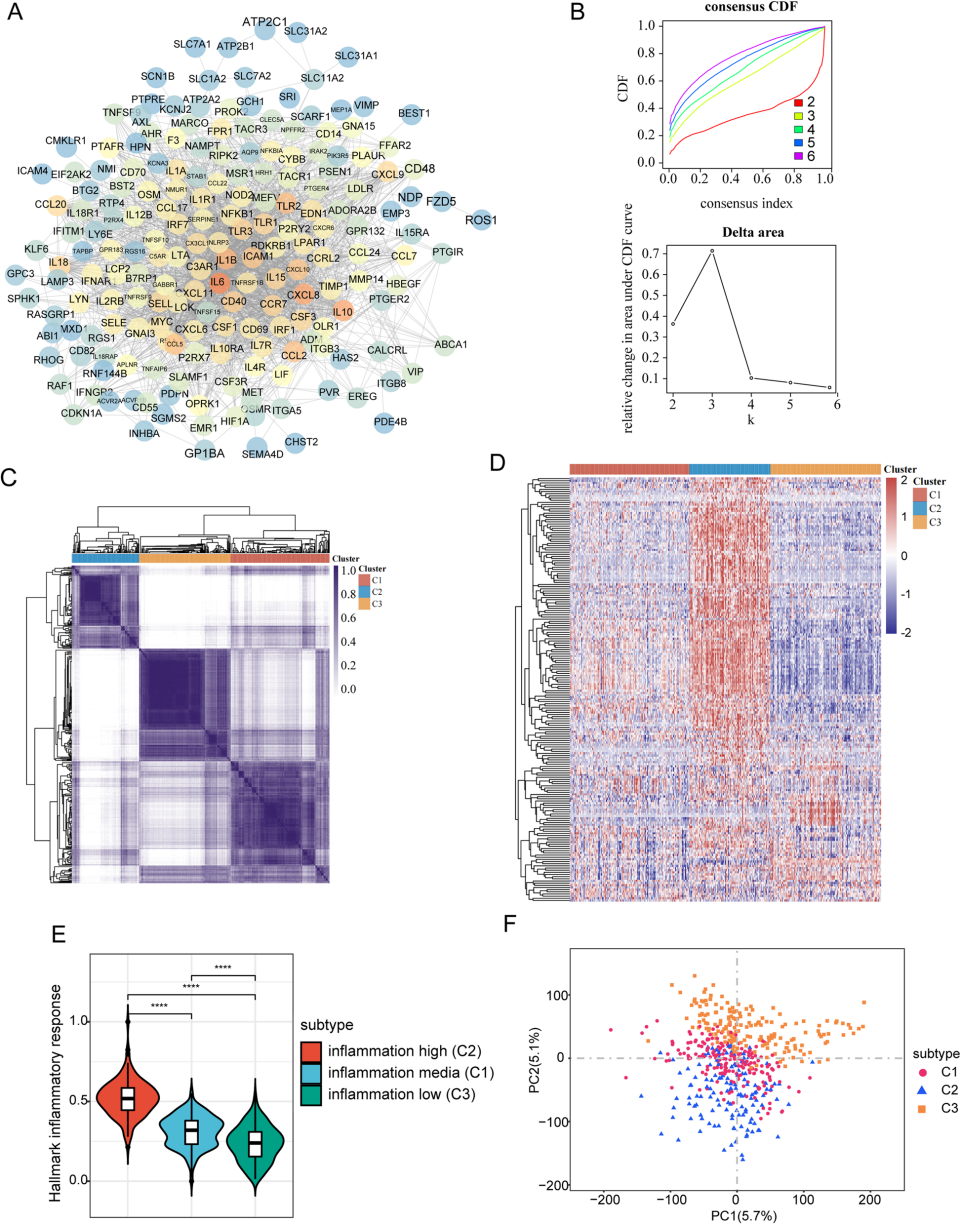
Identification of three inflammation subtypes in LGGs. **A** Protein–protein interactions among 200 inflammation response genes. **B** Delta area curve of consensus clustering. **C** Heatmap depicting consensus clustering solution (*k* = 3) for 200 genes in 509 samples. **D** Heatmap of 200 inflammation response genes expression in different subgroups; red represents high expression, and blue represents low expression. **E** Violin plots indicating the differences in these subtypes. **F** Principal component analysis plots. ****P < 0.0001

The inflammation-related gene set was obtained from gene-set enrichment Analysis (HALLMARK_INFLAMMATORY_RESPONSE). We used the STRING database to perform PPI network analysis on these inflammation-related genes in order to fully comprehend their interactions with one another (Fig. 1A). Subsequently, we identified the LGG inflammation-based clusters utilizing consensus clustering. After k-means clustering, we identified 3 clusters within the TCGA cohort that showed different expression patterns of inflammation genes (Fig. 1B and C). The expression levels of inflammation genes varied among different clusters. Specifically, clusters C2 demonstrated the highest levels of inflammation genes expression. In contrast, clusters C3 were found to have the lowest expression levels, and clusters C1 showed medium levels (Fig. 1D).

Using the ssGSEA method, we also quantified the inflammatory response score of each patient. The result revealed that patients stratified into clusters C2 present the highest inflammatory response score followed by C1 and C3 (Fig. 1E). Hence, we designated clusters C2 as an inflammation-high subtype, clusters C3 as an inflammation-low subtype, and clusters C1 as an inflammation-mid subtype. Subsequently, to compare the transcriptional patterns of the various inflammatory subtypes, the principal component analysis (PCA) was conducted. In general, PCA illustrated that the samples from the three clusters were highly isolated from one another, which indicated distinct transcriptional profiles among these subtypes (Fig. 1F).

We further validated the repeatability of inflammation-based classification in three large independent sample cohorts (CGGA, *n* = 575, and Rembrandt, *n* = 121). Similarly, patients in CGGA and Rembrandt cohort can be stratified into inflammation-low, inflammation-mid, and inflammation-high subtypes (Additional file 3).

### Patients stratified into different inflammation subtypes presented variant prognosis and clinicopathologic features

**Fig. 2:**
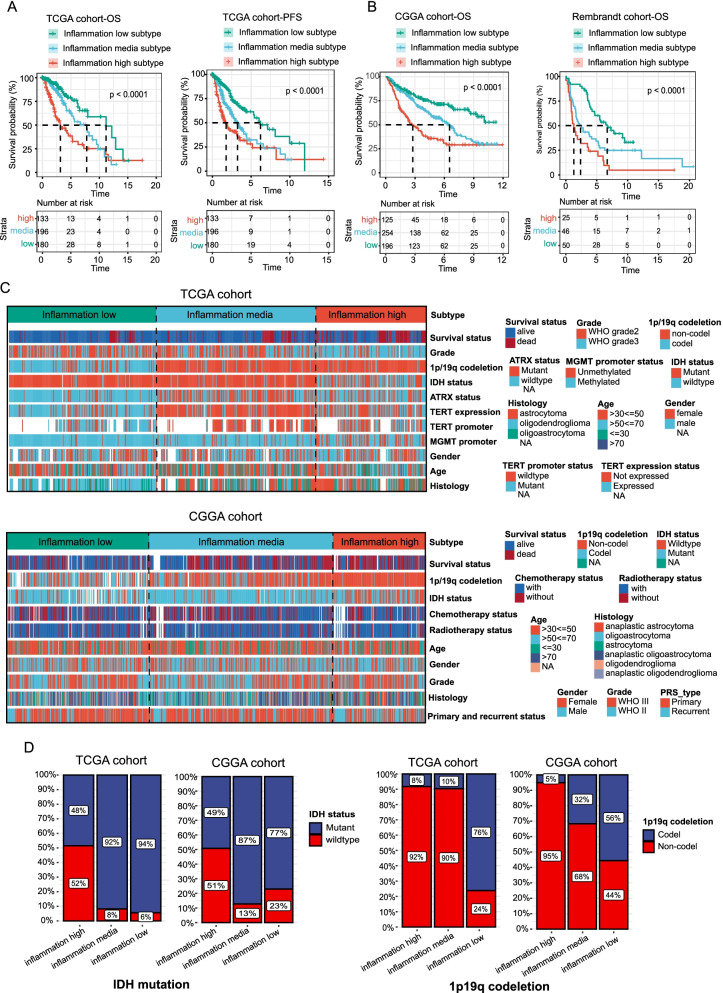
Difference of prognosis and clinicopathologic features among the inflammation subtypes. **A** and **B** Kaplan-Meier overall survival curves for patients assigned into inflammation-low, -mid, and -high subtypes in TCGA (**A**), CGGA, and Rembrandt cohort (**B**). **C** Heatmap presenting the clinicopathologic features of these subtypes. **D** 1p19q codeletion and IDH1 mutation frequency among these subtypes

Previous studies showed that inflammatory responses play decisive roles in the tumor development of glioma. Survival analyses confirmed that these inflammation-based subtypes had specific clinical outcomes, which was consistent with the available data. In general, the inflammation-high subtype presented a dismal prognosis with the shortest overall survival time and progress-free survival (Fig. 2A). In contrast to the inflammation-high subtype, the inflammation-low subtype was associated with the most favorable clinical outcomes among the three subtypes. These findings were additionally validated in CGGA and the Rembrandt cohort (Fig. 2B).

We further compared the clinicopathologic features of the three subtypes. Patients stratified into inflammation-high subtypes were associated with high mortality, unmethylated MGMT promoter status, IDH wild-type status, 1p19q non-codeletion status, WHO III grade, and astrocytoma histology. Conversely, inflammation-low subtypes mainly included low mortality, methylated MGMT promoter status, IDH mutant status, 1p19q codeletion status, WHO II grade, and oligodendroglioma histology (Fig. 2C and D).

### Inflammation-based subtypes are associated with distinct tumor microenvironments (TME)

**Fig. 3:**
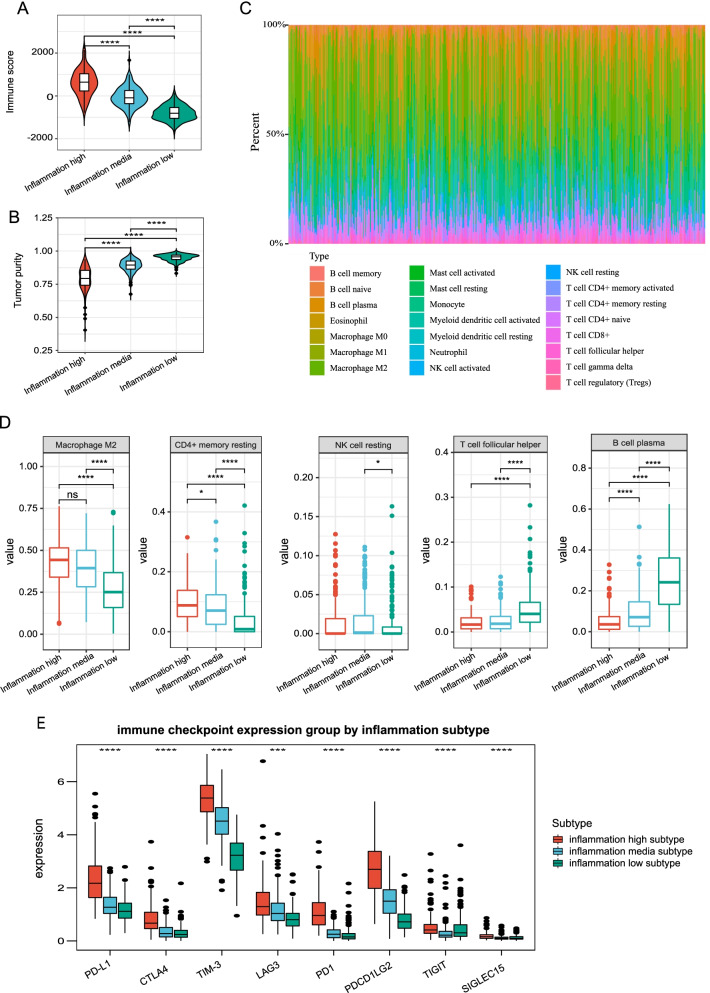
Inflammation-based subtypes are associated with distinct tumor microenvironment. **A** and **B** Violin plots showing the median, quartile, and kernel density estimations for each immune score (**A**) and tumor purity score (**B**). **C** Relative proportion of immune infiltration in LGG samples. **D** and **E** Boxplots representing the differential distribution of immunoreactive, immunosuppressive cells (**D**) and immune checkpoints (**E**) in the various inflammation subtypes

TME composition has been shown to be significantly altered by inflammation, which has a strong impact on immune cells in particular. We examined the TME compositions among different subtypes. Briefly, the immune score shared a gradual decrease from the inflammation-high to the inflammation-low subtypes (Fig. 3A), whereas tumor purity demonstrated a gradual increase (Fig. 3B). These indicated that inflammation high was infiltrated with a higher level of immune cells.

Next, the CIBERSORT method was performed to determine the immune heterogeneity among these subtypes. Figure 3C summarizes the landscape of 22 different immune cell infiltrations. In detail, patients with inflammation-high subtype exhibited substantially greater levels of immunosuppressive cells (M2-type macrophages) and resting immune cells (e.g., resting CD4 memory cell and resting NK cells) but significantly lower proportions of T-cell follicular helper and B-cell plasma (Fig. 3D). Besides, most of the immune checkpoint was elevated in the inflammation-high subtype. Conversely, an inverse pattern was revealed in the inflammation-low subtype (Fig. 3E). These findings illustrated that the immunosuppressive cells, inactivated NK cells, and enhanced expression of an immune checkpoint may drive the immunosuppressive microenvironment of inflammation-high subtype.

**Fig. 4:**
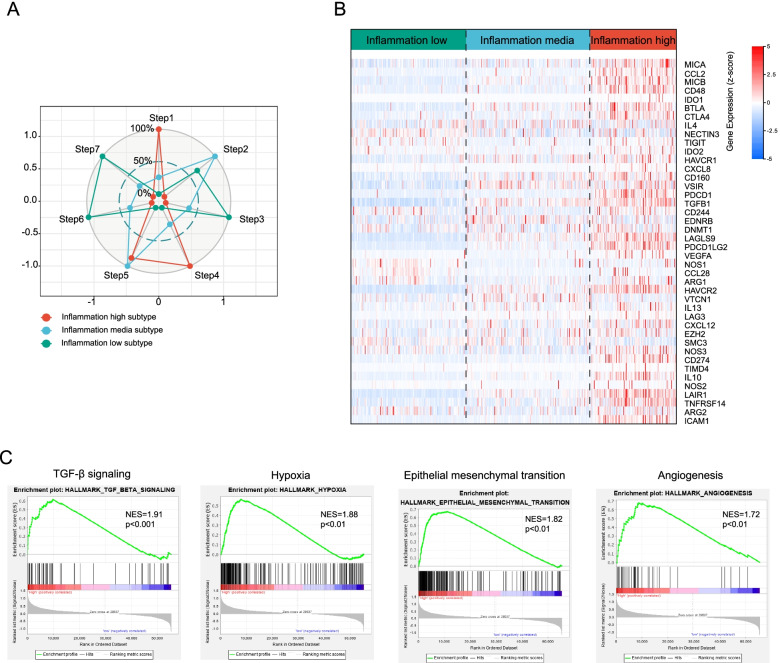
Estimation of anticancer immune activity among inflammation subtypes. **A** Anticancer immune activity of the seven-step cancer-immunity cycle. **B** Heatmap presenting genes expression involved in the negative regulation of the immune processes. **C** GSEA analysis reveals the underlying biological processes correlated with inflammation subtypes

Antitumor immunity may be interpreted as seven sequential processes collectively referred to as the “cancer-immunity cycle” (Additional file 4). We evaluated the anticancer immunological function of the seven-step cancer-immunity cycle in three subtypes using TIP (a web service for determining tumor immunophenotype profiling). Although inflammation-high subtype presented the high activity of step 1 (antigen release from tumors), step 4 (T cells transfer to tumors), and step 5 (immune cells infiltration into tumors), the great attenuation of step 6 (tumor cell detection by T cells), and step 7 (tumor cells apoptosis) was observed (Fig. 4A). However, inflammation-high subtype was associated with enhanced activity of step 6 and step 7 but a restrain of step 1, step 4, and step 5 (Fig. 4A). These indicated that mitigation of immunosuppressive microenvironment in inflammation-high subtype and amelioration of immune cell infiltration in inflammation-low subtype might contribute to good clinical outcomes in LGGs. Besides, genes that participated in the negative modulation of the immune processes were predominantly upmodulated in inflammation-high subtype followed by inflammation-mid subtype and low inflammation-low subtype (Fig. 4B).

Moreover, we analyzed the underlying pathways correlated with the inflammation subtypes. GSEA-enrichment analysis revealed that inflammation-high subtype had a substantially enriched negative modulation of the immune pathways, including TGF-β signaling, hypoxia, epithelial-mesenchymal transition, and angiogenesis (Fig. 4C).

These findings suggested that patients with inflammation-high subtype are prone to developing an immunosuppressive microenvironment that is characterized by the up-modulation of immunomodulatory cytokines, immune checkpoints expression, and immunosuppressive cell infiltration, which may ultimately contribute to the dismal prognosis.

### Somatic mutations landscape in inflammation-high, inflammation-mid, and inflammation-low subtypes

**Fig. 5:**
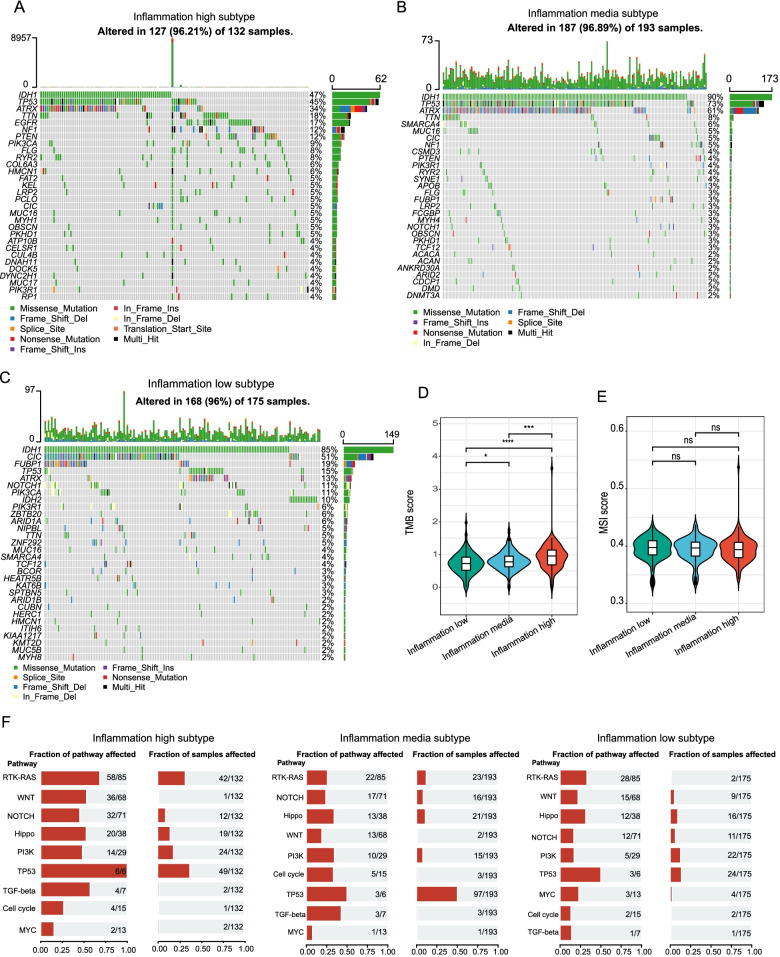
Comparison of somatic mutations among different LGG subtypes. **A–C** Oncoprint visualization of the top 30 most frequently mutated genes in inflammation high subtype (**A**), inflammation media subtype (**B**), and inflammation low subtype (**C**). **D** and **E** Violin plots presenting the TMB score (**D**) and MSI score (**E**) of these subtypes. **F** The mutation frequencies of nine common oncogenic pathways in each of these three subtypes

We discovered that the somatic mutation patterns of these subtypes were different. Even though IDH is the most common mutation, the relative rates of IDH mutations vary across various subtypes. Inflammation-low and inflammation-mid subtypes were found to have an increased IDH1 mutation frequency, which accounted for 85% and 90% of the total mutations while only 47% mutations for inflammation-high subtype (Fig. 5A–C). Besides, inflammation-mid subtypes presented the highest frequency of TP53 mutations (73%) followed by inflammation-high (45%) and inflammation-low subtypes (15%). Moreover, the tumor mutation burden score shared a gradual increase from inflammation-low to inflammation-high subtypes (Fig. 5D), while no significant differences were observed in terms of microsatellite instability (Fig. 5E).

Subsequently, we examined the mutation frequency in 9 major oncogenic pathways in each one of those subtypes. Our results revealed that most of the oncogenic mutated pathways were detected in inflammation-high and -mid subtypes, including RTK-RAS, PI3K, TP53, Notch, and Hippo pathways (Fig. 5F). Notably, these oncogenic mutated pathways were rarely detected in the inflammation-low subtype.

### Establishment and verification of the inflammation-related prognostic signature

**Fig. 6:**
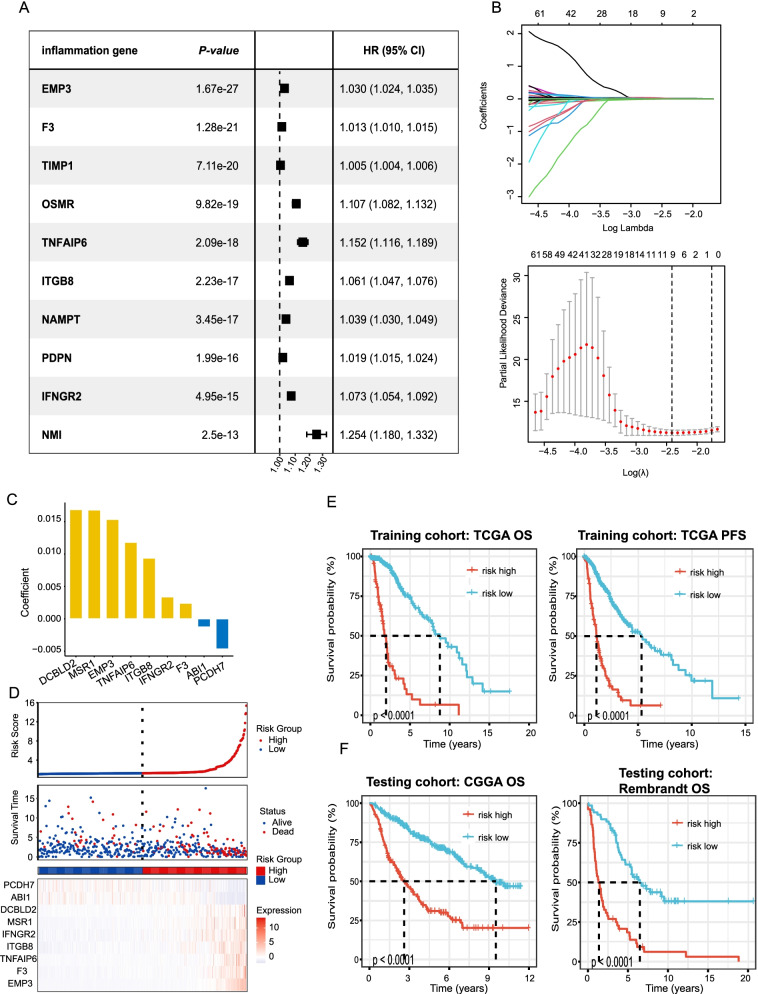
Construction and validation of the inflammation-related prognostic signature. **A** Univariate cox analysis of 200 inflammation genes associated with overall survival. Top ten genes with most significant *p*-value are presented. **B** Lasso Cox analysis uncovered nine genes most associated with OS. **C** The coefficient of the nine genes identified by Lasso Cox analysis. **D** Risk scores distribution, survival status of each patient, and heatmaps of prognostic nine-gene risk signature. **E** and **F** Kaplan-Meier curves for patients with high- or low-risk scores in TCGA training cohort (**E**), CGGA testing cohort, and Rembrant cohort (**F**)

We further created a prognostic model depending on inflammation genes. One-hundred thirty-nine of the 200 inflammation genes were identified as having a substantial association with the patients’ OS according to the results of the Cox univariate analysis. Figure 6A summarized the top ten genes with the most significant *p*-value. As depicted in Fig. 6B, 139 inflammation genes identified by Cox univariate analysis were evaluated and chosen for predicting the prognostic value of the model in the LASSO regression analysis. The development of the risk-score model was achieved according to the following equation: risk score = (0.0153) × EMP3 + (0.0024) × F3 + (0.0118) × TNFAIP6 + (0.0093) × ITGB8 + (0.0034) × IFNGR2 + (0.0168) × MSR1 + (0.0169) × DCBLD2 + (−0.0013) × ABI1 + (−0.0047) × PCDH7 (Fig. 6C). Additionally, we examined the relationship between risk score and survival status. As illustrated in Fig. 6D, our findings revealed that in the low-risk cohort, the number of alive statuses substantially elevated in contrast with that of the high-risk cohort. The prognostic value regarding the risk model was further determined utilizing Kaplan–Meier analysis. Overall, the high-risk score was associated with the unfavorable OS and PFS in the TCGA training cohort (Fig. 6E), which was additionally verified by the CGGA and Rembrandt testing cohort (Fig. 6F).

### The inflammation risk signature has significant predictive value for prognosis evaluation

**Fig. 7:**
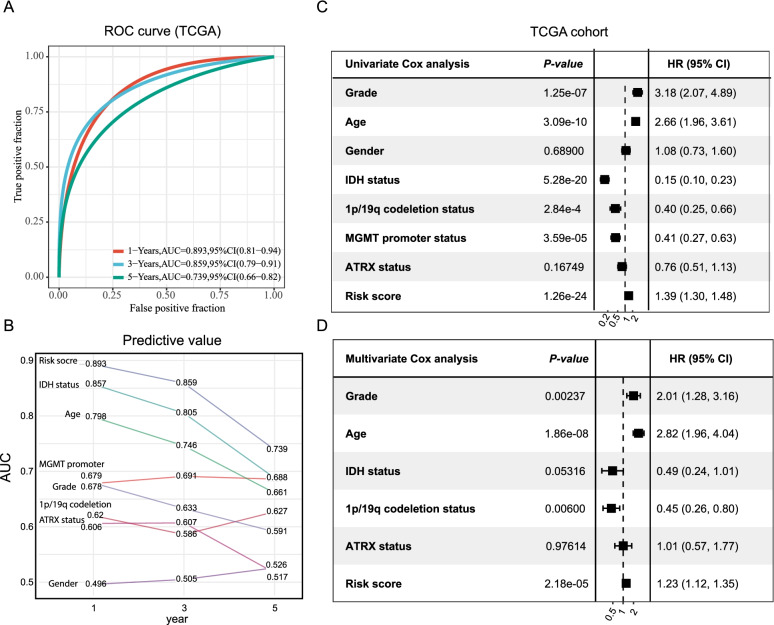
Prognostic value of the inflammation-related risk signatures in LGG. **A** ROC curve showing the predictive value of inflammation risk signature for 1-, 3-, and 5-year survival rates. **B** Comparison of predictive value between inflammation risk signature and clinicopathologic features. **C** and **D** Univariate Cox (**C**) and multivariate Cox analyses (**D**). Evaluating the independent prognostic value of the inflammation risk signature in terms of OS

We conducted a receiver operating characteristic (ROC) curve to estimate the prediction effectiveness of the inflammatory risk signature in terms of 1-, 3-, and 5-year survival rates. Moreover, the 1-, 3-, and 5-year areas under the ROC curve (AUC) were 0.893, 0.859, and 0.739, respectively, demonstrating a strong predictive significance (Fig. 7A). We also compared the prognostic efficiency of the inflammation risk signature based on clinical characteristics in LGG, such as 1p19q status, grade, IDH status, gender, MGMT promoter status, age, and ATRX status. The results demonstrated that inflammation risk presented the best performance in predicting the prognosis compared to other clinical characteristics (Fig. 7B).

Multivariate and univariate Cox analyses were subsequently conducted to estimate the independent prognostic significance of inflammation risk signature with respect to the OS. As illustrated in Fig. 7C, the findings from the univariate analysis illustrated that high inflammation risk score was considerably associated with an unfavorable OS. Other parameters related to unfavorable OS included 1p19q status, IDH status, grade, age, and MGMT promoter status. Figure 7D depicts the findings from multivariate analysis, which illustrated that high inflammation risk score exhibited an independent link to a considerably unfavorable OS, implying that it could independently act as a prognostic predictor for LGG patients (Fig. 7D).

### Validation of inflammation genes expression pattern via scRNA-seq analysis

**Fig. 8:**
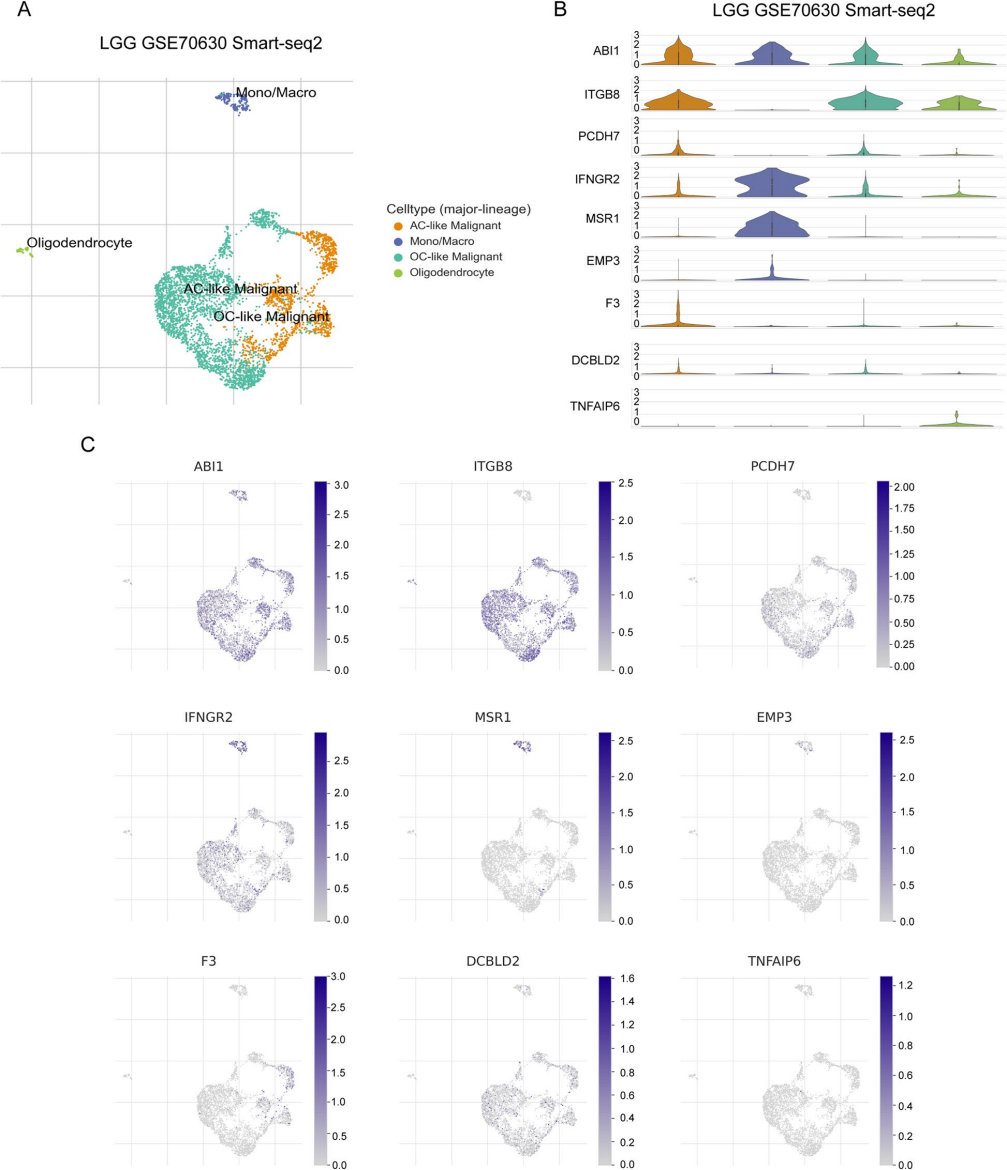
scRNA-Seq reveals inflammation genes expression patterns. **A** UMAP plots showing major cell subsets identified by 10× genomics. **B** and **C** Violin plots (**B**) and UMPA (**C**) plots showing different expression patterns of inflammatory response genes

To confirm further that the detailed type of cells expressing these inflammation genes constituted the risk signature in the TME, we analyzed LGG scRNA-seq utilizing data from GSE70630. A total of 4 cell clusters were detected via uniform manifold approximation and projection (UMAP), namely AC-like malignant cells, monocyte-macrophages, OC-like malignant cells, and oligodendrocyte (Fig. 8A). The results showed that ABI1, ITGB8, and PCDH7 were predominantly expressed in malignant cells, while IFNGR2, MSR1, and EMP3 were predominantly expressed in monocyte macrophages (Fig. 8B and C). Besides, F3, DCBLD2, and TNFAIP6 were detected at low level in both non-tumor cells and tumor cells.

## Discussion

In the present study, we were interested in identifying LGG subtypes according to their inflammatory responses. Our results demonstrate that LGG might be classified into inflammation-high, -mid, and -low subtypes with distinct clinicopathologic features, prognostic, and tumor microenvironment. The findings in the present study demonstrated that this kind of classification was repeatable as well as predictable. In general, the inflammation-high subtype presents a dismal prognosis with the immunosuppressive microenvironment and high frequency of oncogene mutation. In contrast, the inflammation-low subtype was associated with the most favorable clinical outcomes with the immunoreactive microenvironment among the three subtypes. Moreover, we develop and validate an inflammation-related prognostic model, which presents strong power for prognosis assessment.

Historically, the central nervous system was defined as an immune privilege. This understanding was based on the presence of tight junctions in the blood-brain barrier and the absence of a classic lymphatic drainage system. However, this notion of immune privilege has been revised since the discovery of a functional lymphatic system in mice along the dural sinuses [18, 19]. Currently, it has been established that functional lymphatic vessels exist in the CNS, as well as antigen-presenting cells (APCs) of many types, including microglia, macrophages, astrocytes, and classic APCs such as dendritic cells (DCs). Moreover, in certain brain tumors, the blood-brain barrier is often damaged, allowing the infiltration of multiple immune cell types from the peripheral circulation [20]. Although brain tumors present relatively low tumor-infiltrating T cells indicating immunologically “cold,” the majority of immune cells are macrophages, often comprising up to ~30% of the tumor mass [21, 22]. Current studies show that TAMs in glioma are predominantly of the immunosuppressive M2 subtype and play an immunosuppressive role via upregulating the expression of PD-L1 [23, 24]. Besides, chronic inflammation meditated by macrophages M2 is proved to drive glioma growth [25]. In our analysis, patients with inflammation high exhibited substantially elevated levels of macrophages M2, which may account for the high expression of immune checkpoint molecular in inflammation-high subtype.

The tumor microenvironment performs an instrumental function in the occurrence and progression of glioma. A glioma TME is comprised of immune cells, endothelial cells, tumor cells, and a range of cytokines released by the cells. The immune cells found in glioma TME include dendritic cells, microglia, natural killer cells, myeloid-derived suppressor cells, regulatory T cells, T lymphocytes, and macrophages. These cells interface with tumor cells and contribute to the regulation of immunological actions inside the TME [26]. In the glioma TME, the most multifunctional cells group is the glioma-associated microglia and macrophages (GAMs) [27]. When exposed to a variety of microenvironments, GAMs exhibit high plasticity and may undergo polarization into a number of distinct phenotypes. M1 and M2 are the two phenotypes of activated GAMs that have been identified so far. M1 and M2 have diametrically opposed functions. Specifically, the M1 phenotype possesses antitumor properties, while the M2 phenotype possesses immunosuppressive properties and produces cytokines such as EGF, IL-1B, IL-6, and TGF-B to stimulate the growth, invasion, and migration of gliomas by promoting the formation of tumor-related blood vessels and tumor metastasis [23, 28–30]. In our study, patients with different inflammation subtypes presented distinct tumor microenvironments. Patients with inflammation-high subtype have a higher likelihood of developing immunosuppressive microenvironment that is characterized by the up-modulation of immune checkpoints expression, immunosuppressive cytokines, and immunosuppressive cell infiltration, which may ultimately contribute to the dismal prognosis. Notably, patients with inflammation high exhibited substantially elevated levels of macrophages M2.

Despite a multiplicity of clinical trials investigating immune checkpoint inhibitors, the potential predictive biomarkers are still uncertain in glioma. Recently, three studies have focused efforts on in-depth analysis of glioblastoma tissue from patients treated with immune checkpoint inhibitors (ICIs) therapy [31–33]. The results show increased expression of chemokine transcripts, IFNγ-related genes, higher infiltration of immune cells, and increased diversity of TCR clones among tumor-infiltrating lymphocytes, supporting an immunomodulatory effect of the ICIs therapy. However, limited by sample size, these finds need verification with large sample sizes. In our analysis, inflammation-high LGG patients were associated with high immune infiltration and may be potentially sensitive to the current ICIs therapy. Nevertheless, it should be noted that the association between inflammation subtypes and immunotherapies of LGG requires further validation in vitro or in vivo. Our findings should be interpreted with this limitation in mind.

It is important to note that gliomas are significantly diverse, with several subtypes. A precise approach for classifying glioma has been developed as a result of the finding of numerous critical genetic markers, the most notable of which being IDH mutations and the 1p/19q deletion [8, 11, 34–36]. This method has strong prognostic values. Several large randomized studies, including the initial retrospective series and successive retrospective analyses, have shown that 1p/19q deletion is a powerful prognostic and predictive indicator in LGG. Here, we compared the predictive efficiency of inflammation risk signature with 1p/19q deletion, IDH mutation, MGMT promoter status, and ATRX status. The results demonstrated that inflammation risk presented the best performance in predicting the prognosis compared to other clinical characteristics.

To conclude, we developed a new glioma classification system on the basis of the inflammatory subtype of the tumor. This classification produced meaningful results in evaluating patients’ prognoses and the tumor microenvironment.

## Supplementary Information

**Additional file 1.** The clinical information of LGG patients obtained from TCGA, CGGA, and GEO database.

**Additional file 2.** Gene list of hallmark inflammatory response.

**Additional file 3.** Validation of the repeatability of inflammation-based classification in CGGA (A), and Rembrandt (B) cohort.

**Additional file 4.** Illustration of the seven-step cancer-immunity cycle. The seven-step cancer-immunity cycle encompasses the production of tumor antigens (step 1), presentation of tumor antigen (step 2), priming, and activation (step 3), T cells transfer to tumors (step 4), immune cell infiltration into tumors (step 5), tumor cell detection by T cells (step 6), and tumor cells apoptosis (step 7).

## Data Availability

The datasets generated and/or analyzed during the current study are available in the TCGA, CGGA, and GEO repository (https://www.cancer.gov/tcga;http://www.cgga.org.cn/; https://www.ncbi.nlm.nih.gov/geo/).

## Abbreviations

CTLs: Cytotoxic T lymphocytes
CGGA: Chinese Glioma Genome Atlas
GAMs: Glioma-associated microglia and macrophages
ICIs: Immune checkpoint inhibitors
LGG: Lower-grade glioma
OS: Overall survival
PCA: Principal component analysis
PPI: Protein-protein interaction
ROC: Receiver operating characteristic
ssGSEA: Single-sample gene-set enrichment analysis
TCGA: The Cancer Genome Atlas
TME: Tumor microenvironments
Tregs: T cells regulatory
TISCH: Tumor immune single-cell hub
UMAP: uniform manifold approximation and projection
