# Supplementary figures and images for: Identification of molecular subtypes based on inflammatory response in lower-grade glioma
Source: Inflamm Regen. 2022 Oct 1;42:29. doi: 10.1186/s41232-022-00215-9 (PMC9526248; doi:10.1186/s41232-022-00215-9)

A

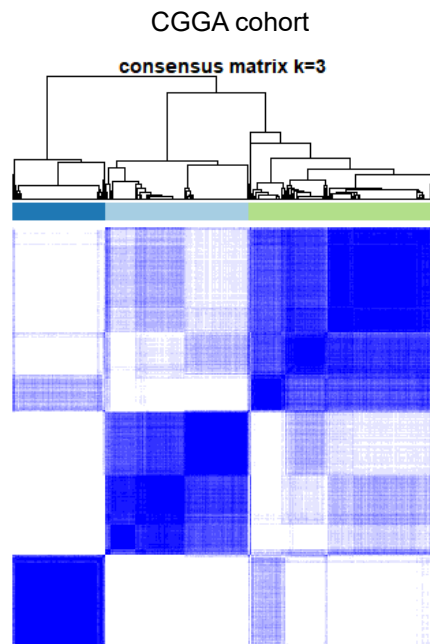

B

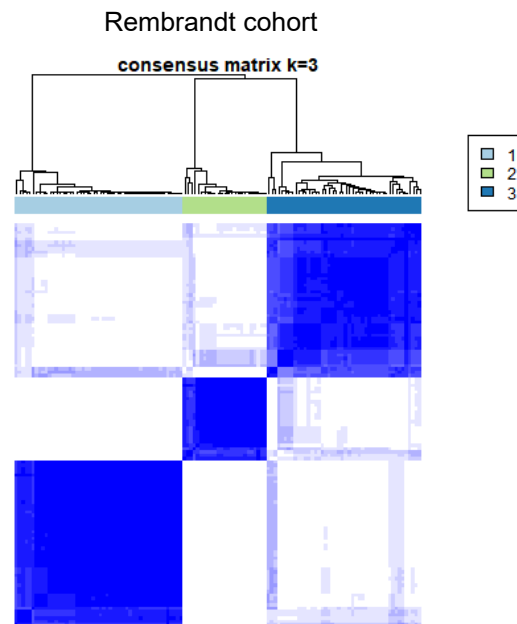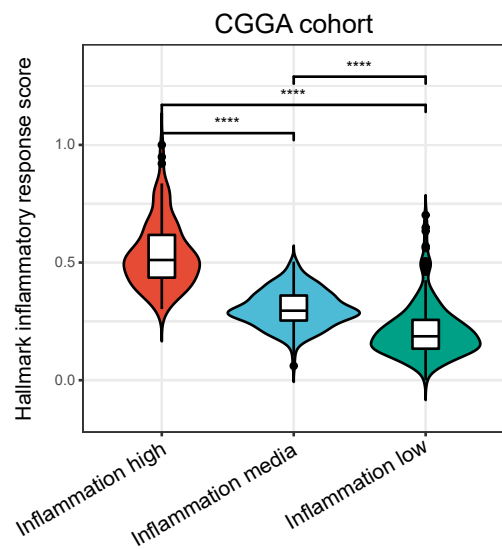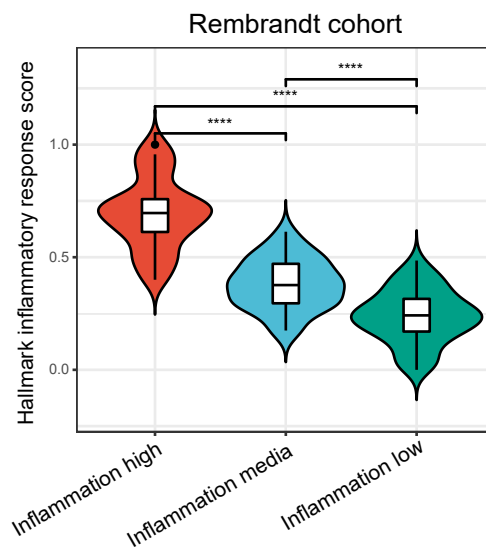

Supplement: Supplementary file 3 — Additional file 3. Validation of the repeatability of inflammation-based classification in CGGA (A), and Rembrandt (B) cohort. [file 41232_2022_215_MOESM3_ESM.pdf]

A

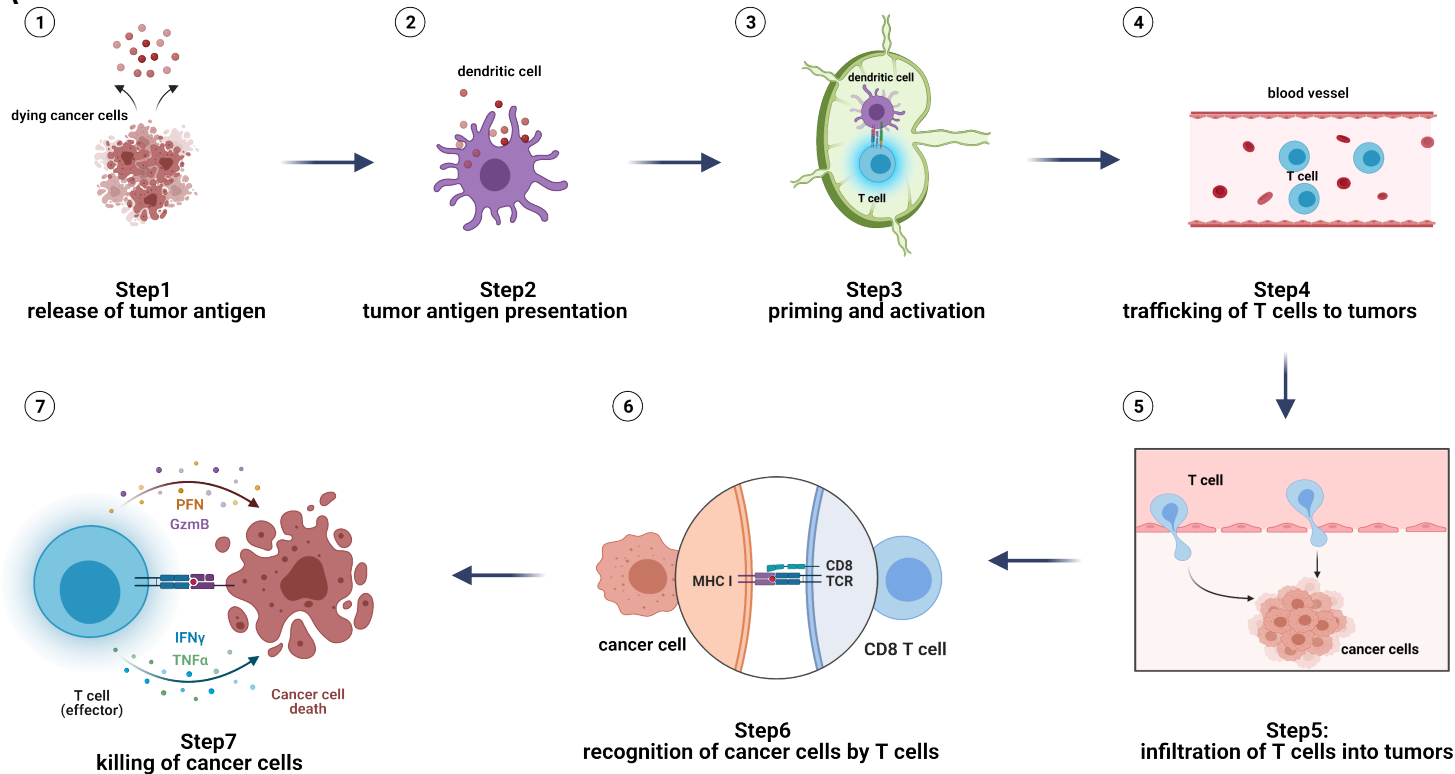

Supplement: Supplementary file 4 — Additional file 4. Illustration of the seven-step cancer-immunity cycle. The seven-step cancer-immunity cycle encompasses the production of tumor antigens (step 1), presentation of tumor antigen (step 2), priming, and activation (step 3), T cells transfer to tumors (step 4), immune cell infiltration into tumors (step 5), tumor cell detection by T cells (step 6), and tumor cells apoptosis (step 7). [file 41232_2022_215_MOESM4_ESM.pdf]
